# Employers’ experience of employees with cancer: trajectories of complex communication

**DOI:** 10.1007/s11764-017-0626-z

**Published:** 2017-07-14

**Authors:** C. M. Tiedtke, B. Dierckx de Casterlé, M. H. W. Frings-Dresen, A. G. E. M. De Boer, M. A. Greidanus, S. J. Tamminga, A. E. De Rijk

**Affiliations:** 1 Department of Social Medicine, Faculty of Health Medicine and Life Sciences (FHML), Maastricht University, Maastricht, The Netherlands; 2 Department of Public Health & Primary Care, Academic Centre for Nursing and Midwifery, KU Leuven, Leuven, Belgium; 3 Academic Medical Center, Public Health Research Institute, University of Amsterdam, Amsterdam, The Netherlands

**Keywords:** Return to work, Workplace support, Qualitative, Communication, Cancer, Employer perspective

## Abstract

**Purpose:**

Remaining in paid work is of great importance for cancer survivors, and employers play a crucial role in achieving this. Return to work (RTW) is best seen as a process. This study aims to provide insight into (1) Dutch employers’ experiences with RTW of employees with cancer and (2) the employers’ needs for support regarding this process.

**Methods:**

Thirty employer representatives of medium and large for-profit and non-profit organizations were interviewed to investigate their experiences and needs in relation to employees with cancer. A Grounded Theory approach was used.

**Results:**

We revealed a trajectory of complex communication and decision-making during different stages, from the moment the employee disclosed that they had been diagnosed to the period after RTW, permanent disability, or the employee’s passing away. Employers found this process demanding due to various dilemmas. Dealing with an unfavorable diagnosis and balancing both the employer’s and the employee’s interests were found to be challenging. Two types of approach to support RTW of employees with cancer were distinguished: (1) a business-oriented approach and (2) a care-oriented approach. Differences in approach were related to differences in organizational structure and employer and employee characteristics. Employers expressed a need for communication skills, information, and decision-making skills to support employees with cancer.

**Conclusions:**

The employers interviewed stated that dealing with an employee with cancer is demanding and that the extensive Dutch legislation on RTW did not offer all the support needed. We recommend providing them with easily accessible information on communication and leadership training to better support employees with cancer.

**Implications for cancer survivors:**

• Supporting employers by training communication and decision-making skills and providing information on cancer will contribute to improving RTW support for employees with cancer.

• Knowing that the employer will usually be empathic when an employee reveals that they have been diagnosed with cancer, and that the employer also experiences difficulties and dilemmas, might lower the threshold to discuss wishes regarding disclosure, communication, and work issues.

• The interests of employer and employee in relation to RTW are interrelated; both have responsibility and a role to play, and are in need of support.

## Purpose

Worldwide, there are over 14 million new cases of cancer each year [1]. In Europe, over three million people receive a cancer diagnosis each year [2]. Due to improvements in treatment and survival rates, return to work (RTW) after cancer is of increasing importance for survivors, for their employers and for society at large [3, 4]. Thus far, RTW research has focused on the perspectives of the cancer survivors themselves [5]. Research has clearly shown the importance of a supportive employer to achieve RTW for this group [6–11]. These studies led to the conclusion that employers need to do more to support employees with cancer.

However, a review of employers’ perspectives shows that there is barely any research on their experiences in supporting RTW of their employees with cancer. A Belgian study has shown that employers struggle while supporting their employees with breast cancer [12]. Belgian employers expressed an urgent need for assistance as they experience intense involvement and concern, as well as high levels of uncertainty and many specific dilemmas during their employees’ RTW process. They struggle with employee privacy, conflicting interests of the employee and organization, and conflicting employer roles (e.g., when to take a more personal or a more professional role) [12]. Moreover, not every employee with cancer survives their illness, which might be an additional burden for organizations. Feuerstein et al. [13] reported that employers in the USA do not know how to retain qualified employees with chronic conditions. Employers in this study also expressed a need for assistance. McKay et al. [14] identified a lack of knowledge among Australian managers on how to respond appropriately, which influenced RTW outcomes. Amir et al. [10] concluded that UK employers need to be provided with training, support, and resources to help them facilitate employment and job retention of employees diagnosed with cancer. Other studies included employers as one of the stakeholders investigated regarding RTW needs [15, 16].

The aim of the current study is to consider the experiences of employers in the Dutch legal context. In the Netherlands, the employer is the most important stakeholder in the RTW process and has a relatively large financial and practical responsibility. Employers have to provide payment of at least 70% of the income during sickness absence (but do not pay social premiums for sickness absence benefits, as is the case in many other countries) and are legally obliged to support RTW during 2 years, in collaboration with an occupational physician [17]. After 2 years of sick leave, the public Employee Insurance Agency (EIA) assesses employees for disability benefit (http://www.uwv.nl/overuwv/english). Thus, Dutch employers feel a direct responsibility to achieve (gradual) RTW to the same or another job, and have to accommodate employees returning to work. The EIA checks whether the employer has done what is necessary to achieve RTW and is allowed to sanction an employer if they have not done so. Furthermore, if an employer has decided not to pay premiums for partial disability pension, he or she is responsible for payment of the partial disability pension of his or her employee for a period of 10 years. Full disability pension is always paid by the EIA. The Dutch context, with a clear focus on activation of the work-disabled rather than solely income protection, thus guarantees extensive employer involvement in RTW for employees [18]. This makes the Dutch context interesting for studying employers’ perspectives on employees with cancer.

The purpose of the current study is to gain insight into Dutch employers’ experiences with employees with cancer, with an emphasis on how they experience RTW for cancer survivors. This insight is needed to be able to meet employers’ need for support regarding RTW. The research questions are as follows: (1) How do employers experience their role and responsibility in supporting the RTW process of employee(s) with cancer? (2) What do these experiences imply for employers’ need for support?

## Methods

### Design

To gain an in-depth understanding of employers’ experience, a qualitative research design (Grounded Theory approach) was chosen [19], using the Qualitative Analysis Guide of Leuven (QUAGOL) [20]. Semi-structured interviews were held (CT) to obtain intricate details about feelings, thought processes, and experiences related to real-life situations. We chose to conduct individual interviews to provide a safe environment, so the participants felt that they could talk freely about sensitive information.

### Sample

Participants were recruited by convenience and purposive sampling. We searched for representatives of employers from for-profit and non-profit organizations (small, medium, and large companies). We also adjusted for recruitment of supervisors and small (for-profit) organizations. First, the study was announced on relevant websites (the National Cancer Foundation, employers’ federation, HR union, employers’ network), which yielded three participants (*n* total = 3). Concurrently, a concise flyer was sent to potential informants in the researchers’ professional and personal network. Whenever informants suggested an employer, they were generally interested in participating. This yielded a further eight participants (*n* total = 11). Next, a well-attended, official employer meeting was visited to contact employers during the lunch break, which yielded five participants (*n* total = 16). One of the potential contacts informed their counselors’ network and offered the opportunity to give a presentation (CT) about the study, which ultimately led to another 11 employers participating (*n* total = 27). Then, the snowball method was used to expand the existing study population. All employers were asked to search for additional participants in their network. This yielded three more participants (final sample = 30). Participants who reacted positively were approached directly by e-mail and/or telephone by the first researcher or an intermediary (occupational physicians, counselor coaches, already interviewed employers). To be included in the study, participants had to be responsible for the employee’s support and return and have immediate, preferably recent (<5 years) involvement in the RTW process of one or more employees with cancer. In addition, they had to be willing to discuss real-life cases (no hypothetical cases) regarding the RTW process of employees with cancer. Written informed consent was obtained from all individual participants included in the study. Ethical approval was deemed not necessary by the Medical Ethics Committee of the Academic Medical Center (Amsterdam) (W15_277).

### Data collection

Data were collected by means of semi-structured interviews with the first author, using a summary of topics as an interview guide (Table 1). This guide was developed based on the research questions and on former experience of the investigators [12] and in consultation with all authors. The participating employers were first asked to describe their recent cases of employees with cancer. Next, they were asked about their experiences and perceptions of the sickness absence and RTW process of each of these employees. Finally, they were asked about their role and needs in the process. Each time, concrete examples of experiences were asked for. As the interviews progressed, adaptations to the guide were made in response to new topics arising. The interviews were audio-recorded, transcribed verbatim, lasted 40–120 min, and were held at the workplace.

**Table 1:** Interview guide

Topic area	Subtopics
Sickness absence (SA) *Challenge to give concrete examples* *Probing and summarizing, refer back to points raised earlier in the interview*	Role Procedure sickness absence (*disclosure*, *medical confidentiality*) Implication of the absence for the work to be done (*replacement*, *organized how*) Support and involvement (*relationship with employee*, *reaction of colleagues*, *home visits*) Advice regarding absence (*from who*, *for who*) Communication (*how*, *about what*, *frequency*) Contact, collaboration, and regular meetings in relation to SA (*occupational physician*, *social medical team*, *social insurance*, *other*) Course of the trajectory (*action plan*) Decision-making (*weighing up pros and cons*) Bottlenecks, dilemmas (*which*, *examples*) Need for support Efficiency (*tips*)
Return to work (RTW) *Challenge to give concrete examples* *Probing and summarizing refer back to points raised earlier in the interview*	Initiatives Expectations Support and involvement (*relationship with employee*, *reaction of colleague*) Contact, collaboration, and regular meetings in relation to SA (*occupational physician*, *social medical team*, *social insurance*, *other*) Advice on RTW (*from who*, *for who*) Course of the trajectory (*action plan*, *what*, *and how*) Decision-making (*weighing up pros and cons*) Communication and decision-making (*how*, *about what*) Work adaptations/modifications (*which*) Bottlenecks, dilemmas (*which*, *examples*) Efficiency (*tips*)

### Analysis

The Qualitative Analysis Guide of Leuven (QUAGOL) was used to analyze the data [20]. The QUAGOL is a theory- and practice-based, systematic, non-rigid guide for analyzing qualitative data, based on Grounded Theory principles [19]. It is characterized by iterative processes of digging deeper, constantly moving between the various stages of the process. First, a case analysis was started. All recorded and transcribed interviews were (re)read thoroughly by experienced qualitative researchers (CT, BDdC, ST, AdR). Individual narratives were written per transcript (CT). Narratives and/or interviews were discussed with (parts of) the team to exchange thoughts (CT, BDdC, ST, MG, AdR). From the start, conceptual reports were made for each narrative (CT, BDdC, AdR) to monitor data saturation, and specifically to capture the essence per interview keeping the integrity of the story. This ensured that variety was taken into account. The reports eventually evolved into a common conceptual scheme representing all interviews (CT). A list of meaningful themes was derived based on all conceptual interview schemes (CT). Second, a cross-case analysis was performed using QSR NVivo 10 (QSR International Pty, Ltd., 2013). An inductive process of interpretation followed, with the purpose of discovering dimensions and characteristics of essential concepts and relationships in raw data, and then organizing these into theoretical explanatory schemes (CT, BDdC, AdR). Preliminary findings were discussed with external, experienced scientists (peer debriefing) to contribute to the trustworthiness of the findings.

## Results

Thirty-three employers were invited. The first 30 participants who agreed to participate were included in the study. One employer declined to participate due to lack of time; another did not react, on second thought. An additional potential HR manager denied having experience with an employee with cancer, although the first researcher was informed otherwise by the company director. The employers had medium-sized (100–500) or large-sized (>500) organizations in the for-profit or non-profit sector in different parts of the Netherlands. Half of them had worked in an organizational role for at least 5–10 years, and on average, two to three employee cases were discussed per employer. The 30 interviews were held in 19 different organizations (7 for-profit, 12 non-profit). In 11 of these organizations (3 for-profit, 8 non-profit), one person was interviewed. In eight organizations (four for-profit, four non-profit), two persons or more were interviewed: the HR manager and one or more line managers (LM). Among the HR managers, two had a slightly different position. One was a social worker and the other a sickness absence coach, both working for the HR department. After 30 interviews, saturation was reached on views, roles, and needs of employer representatives. Table 2 provides a summary of participant characteristics.

**Table 2:** Employer characteristics (*n* = 30)

Characteristic	Number
Gender	
Male	12
Female	18
Age group	
30–39	3
40–49	9
50 and older	18
Position	
Human resource manager (HR) (incl. social worker/absence coach)	10
Line manager (LM)	20
Job experience	
0–5 years	3
5–10 years	6
10–15 years	5
15–20 years	2
20–25 years	3
Unknown	11
RTW experience with employees with cancer in	
<5 years	27
≥5 years	3
Average number of employees with cancer (cases discussed)	2–3
Sector	
For-profit	13
Non-profit	17
Size	
Medium (100–500 employees)	9
Large (>500 employees)	21

The employees who were discussed (*n* = 63) had a large variety of cancer diagnoses and occupations (e.g., nurse, secretary, midwife, chauffeur, psychiatrist) in diverse sectors (e.g., public health, management, logistics). One third had breast cancer, about 12% had lung cancer, and seven employees (11%) suffered from several diagnoses (uterine or prostate cancer combined with other tumors, such as breast, bowel, stomach, pelvis, and/or liver tumors). Half of the employees returned to work, 30% did not. Eleven employees (17%) died before or after (part-time) RTW, within the expected 2 years of RTW support. Table 3 provides a summary of employee characteristics. These characteristics were deduced from the interviews; they were not systematically asked for, because of privacy reasons. Infrequently, employers did not mention diagnoses, positions or RTW outcomes.

**Table 3:** Employee characteristics (*n* = 63)

Characteristic	Frequency
RTW	
Yes	32 (1†)
No	21 (10†)
NY	5
Unknown	5
Cancer diagnosis	
Breast	21
Lung	8
Several cancer diagnoses	7
Unknown	7
Bowel	5
Leukemia	4
Bladder	2
Liver	2
Esophagus	2
Brain	2
Pancreas	1
Skin	1
Prostate	1
Sector	
Factory and logistics	16
Public health sector	15
Administrative sector	9
Managerial staff	9
Cleaning sector	6
Unknown	8

*NY* not yet returned (within first or second year of absence)

### Employer experiences

Supporting an employee after cancer diagnosis appeared to be a *continuous communicative trajectory*, which mainly occurred between the employer and the employee. Several aspects relating to illness and employability were discussed throughout different phases, requiring different communication styles. Communication was the key issue, but employers’ specific approaches varied. Two major types of employer approach emerged: a business-oriented approach (based on employer interests) and a care-oriented approach (based on employee needs). Supporting an employee with cancer was experienced as being complex. The complexity derived from the unpredictable cancer diagnosis and the confrontation with dilemmas, especially in balancing the interests of employee and organization. Variations between employers’ experiences related to individual employer and employee characteristics and to organizational differences.

#### Communication during the trajectory

Supporting an employee with cancer was revealed to involve an intense communicative trajectory, from the moment the employee disclosed their illness until either the period after they returned to work, became disabled for work, or passed away, which required different communication styles. As a common finding, the trajectory was phased (*disclosure and impact on the organization*; *waiting for treatment result and creating openness*; *RTW planning and designing*; *actual return*; *no return and death*), although the pace differed across cases and going back to an earlier phase was not unusual. As the trajectory continued, the employers (and their employee) had to justify all the plans, consultations, and actions to the persons concerned for the Dutch Gatekeeper law to prevent financial sanctions. While complying with the legal and organizational rules, employers weighed up their rights and obligations. In specific situations, they departed from the minimum legal requirements (e.g., in case of a serious prognosis or approaching death of an employee).

##### Disclosure and impact on the organization

The communicative trajectory started with the employees’ disclosure of being diagnosed with cancer. According to the interviewees, handling the emotions was their foremost concern. The employers showed empathy and were full of understanding. They tried to find out how to inform colleagues, in accordance with the employee’s wishes. They sounded out if the employee could possibly stay active at work during treatment and if so, in what way. If not, the timing of required replacement could be discussed. Employers varied in this respect. Some wished not to bother the employee with practical, organizational issues.

“…And even if there had not been any substitute for her, it would not have harmed her. That is impossible. Someone is ill, and that is allowed, it should have no impact on the patient (...) He or she should not be bothered by: ‘well, the department cannot function without me’. For that is very relative…” [Resp. 8, LM]

They communicated with the team about the impact of the employee’s cancer diagnosis, which had, from the perspective of the employer, to be addressed in a serious manner. Communication was experienced as particularly difficult in case of an unfavorable prognosis.

“…As executive you address the group: ‘Guys, listen, I have an unpleasant announcement’. Well, then it gets very silent in the canteen and it lingers for a while. People will call on each other and that should be allowed, one should be tolerant in that case…” [Resp. 19, LM]

“…You cannot make an announcement and say: ‘well, let’s get back to work, and if you have problems, just let me know’. That is not done. You have to allow people to cope with it…” [Resp. 7, LM]

##### Waiting for treatment result and creating openness

After having dealt with the shock of the employee’s cancer diagnosis and the initial decisions, the interviewed employers tended to take distance from the employee, to give him/her some privacy, and to make room for the employee’s concern of being ill and having to undergo treatment.

“…You just give her a break, but she was also too ill. I am not the kind of executive who immediately rushes to the hospital with a bunch of flowers. I will have it delivered, of course (…). But do keep in touch, send some flowers, a card, an app. Do something extra…” [Resp. 26, LM]

They wanted to meet the employees’ wishes. They also felt that it was “not done” to burden an employee with cancer with any obligations at this stage.

“…For I think, those people have enough to worry about. And if you put pressure on them, well, you know, that doesn’t work as I see it. It doesn’t work. Look, it is not the flu…” [Resp. 25, LM]

As the trajectory wore on, the employers tried to stay in contact with the employee to assess the needs, expectations, and interests (on a personal and company level) and to further discuss work consequences and the employee’s employability. Employers mentioned that it was difficult to decide when to discuss employability with the employee.

“…How can I make that negotiable, should I beat about the bush for a while, can we discuss work or is it too early…?” [Resp. 20, HR]

According to the employers, the occupational physician was also contacted to explore suitable RTW options, with the purpose to come to an understanding together with the employee.

“…That I also confront him with it like: ‘what do you think yourself?’ Then, by discussing it, we can come to conclusions about: how do you see it…” [Resp. 13, LM]

The interviews revealed that the employers kept in close touch with the employee to create openness and transparency. A mutual responsibility was felt during this stage, since employers also tended to expect the employee to keep in touch with the department regarding the situation.

“…And he did allow us to keep in touch. And he also kept in touch with the department; to me that was very essential. For that keeps it within reach…” [Resp. 17, LM]

##### RTW planning and designing

The interviews showed that communication proceeded with concrete RTW planning and deciding on the required job adjustments in a thoughtful way. Beforehand, employees were informed about consultations with the occupational physician and other parties involved.

“…When I think that someone can do an alternative type of work, I will have him picked up by a taxi, if need be (…) after all, I am not a doctor, so you check: well this is the situation, this is the job I want to offer, is it justified? And yes, fine, let’s do it…” [Resp. 24, LM]

According to the employers, the legal rights and duties of both employer and employee were also discussed, if these had not already been brought up earlier. Discussing these issues was experienced as difficult, because of the impact on opinions and feelings of both parties (e.g., in case of different views on employability).

“…But one needs the skills to do it well together and explain it well. And if you can’t agree on every aspect, then, to my opinion, you should give a wide berth to find consensus together. And, at some given moment, an employee should be allowed to say: actually I wanted to do this and that…” [Resp. 10, HR]

##### Actual return

If conditions were favorable, the employers felt that they had to invite and help the employee to return to work in phases, with a reduction in working hours, whether or not in an adjusted job. The interviewees preferably tried to reach this goal without pressure, and by meeting the employee’s wishes. The employers said colleagues were informed and asked for their help, especially during the first days after the employee’s return.

“…That they somehow know like, you know, why is someone at home, what is going on. Can or can’t we send a card, or doesn’t he want any contact. And when someone comes back, well, what can we expect from her, what is she going to do, what can and what can’t she do. And sometimes I have the employee write a note, like, well, what would you like the team to know…” [Resp. 16, LM]

However, as the interviewees mentioned, additional adaptations were needed when the job was observably still too tiring. New arrangements (e.g., reduced working hours or a different schedule) had to be made in such cases, in close consultation with the employee.

“…That’s what I mean by customization: also listen to what someone says. And perhaps someone will fail, but then you can register it, like, we think you take a risk, but we will try…” [Resp. 27, LM]

From the perspective of the employers, a serious reduction in performance could take place after the employee had returned. Other (or external) reintegration options were discussed in such cases, in deliberation with other parties involved (the occupational physician, social workers, personnel management), starting with the employee. Employers felt that discussing these options required their full attention.

“…His doctors insisted that he should take much better care of himself. So that called for a different approach: pay much more attention to the fact that he could accept to resign from his work (…) In the end he had another operation, was treated, came back and went through another transition. And that is, to my opinion, the nice side of this kind of processes, that he said: ‘I would very much like to go on working, but I don’t think it is wise to keep doing the full job’…” [Resp. 10, HR]

In case of serious disagreement on RTW options, the employers tended to concentrate on fulfilling the legal duties to avoid being sanctioned by the national social security agency.

“…The compulsory contact moments are unavoidable. For if you don’t follow the rules, then, as an employer, you face financial consequences. Therefore, it is a challenge to find a good balance between obligation versus personal [situation] and the agreements…” [Resp. 11, HR]

##### No return and employee’s decease

As the participants mentioned, disability pension had to be discussed and requested in case of no reintegration after 2 years because of severe health limitations on a permanent basis.

“…I don’t know what the possibilities are, but I don’t think there are many possibilities to keep X working. And not because we don’t want to, in that case we would think of something. But because he can’t. He can’t memorize anything; he is no longer allowed to drive a car, so how could he get here…” [Resp. 22, LM]

In case of an unfavorable prognosis, death was also talked about. It was heartbreaking and distressing for both the employer and the employee, when the end of life appeared to be approaching.

“…Different from other diseases, such a department is closely involved. We see him less often, but you notice people are joining him on, in fact, the road to the end…” [Resp. 7, LM]

According to the employers, communication during this phase took an enormous effort, also because of a lack of existing guidelines.

“…I found it more difficult that at a given point ‘death’ becomes an issue, and then I think: ‘wow, how sad’. But that is no basis for a conversation, like ‘wow you are to be pitied’. But how do I cope with that within our strategy?...” [Resp. 29, HR]

The death of an employee also had a deep impact on the colleagues and team, and called for particular attention and care, according to the employers.

“...During our 25 years’ history, we have never seen the last agony of one of our colleagues. It really deepens the impact. We gave shifts the possibility to attend the funeral: ‘guys stop the work and be off’. And they accepted it gratefully (…) We also have to deal with the finances, which is really unpleasant! (…) And yes, empty the locker, there is still a locker full of personal things (…) We have to contact the family (…) can we leave things with you?...” [Resp. 14, LM]

#### Business- and care-oriented approach

In the communication with their employee, employers had different approaches. Two main types of employer approach emerged throughout the communicative trajectory: a business-oriented approach and a care-oriented approach.

Employers adopting a *business-oriented approach* primarily aimed at reducing the consequences of the absence for the organization and thus at quick recovery and making sure the employee returns to work. The business-oriented approach emphasized interdependence during the whole process. After all, according to these employers, an employment contract existed with formal mutual responsibilities. There was also talk of a relationship of power, which committed both the employer and the employee to take responsibility.

“…From both sides it is a little giving and taking (…) because it can’t just all come from us…” [Resp. 29, HR]

The employee received and the employer provided performance-related pay. This matter could be discussed perfectly well, according to the employers handling things this way. In their opinion, it is no use to only discuss illness and treatment-related topics, except in the case of an unfavorable prognosis. These employers empathized with the employee and paid attention to the future of the organization as a whole.

“…At the same time I work for an organization and it is important that my organization keeps functioning well, so that we all can go on offering jobs to our staff…” [Resp. 11, HR]

They underlined their responsibility as employer and wished to welcome an employee back as soon as possible, as this was the best for both parties’ sakes. Besides, they mentioned that there was only a professional, and no familiar bond with the employee. Consequently, conversations with the employee were preferably held in the office to maintain a professional atmosphere.

“…For we try to focus on mobility, that is our aim, we focus on mobility. If a person is mobile, then the conversation takes place in the office, and not over the phone or at his house, but in the office, if he can come to work. That doesn’t mean that he can start working full time, but the conversation takes place…” [Resp. 20, HR]

Employers who strived for a strong business-oriented approach considered too much personal attention to be disturbing in keeping a professional balance. They would rather strictly follow the steps required by legislation, which forces employers to be involved continuously and take responsibility.

“…This is what we stick to, this is what we expect from you and in exchange you can expect this from us. One should absolutely stick to that explicitness and professionalism, also in coaching this kind of stages…” [Resp. 11, HR]

Employers who adopted a *care-oriented approach* primarily considered the consequences of the diagnosis for the individual employee’s health and the impact on their (continuing) to work in the organization. They put greater value on the impact of a cancer diagnosis on the whole life of their employee. The leading questions were: what are the needs of the employee and how can the employer meet these? Employers related that they took an observing attitude towards the employee. If they thought it was needed, they would put a brake on RTW. To proceed cautiously was the best for both parties’ sakes.

“…That you realize: yes, you are the person in charge, but you are also a person who understands the other one. And not just someone who wants the other one to go back to work, knowingly and willingly…” [Resp. 19, LM]

Some employers went further in supporting their employees with cancer than others. They tended to focus completely on the employee’s interest, leaving out the employer’s interest to find suitable and adapted solutions.

“…We wish everyone here well. And at times we set aside the company’s interest, when we think that that is the right solution …” [Resp. 22, LM]

We also saw combinations of both approaches, as many employers strived to seek a balance. Some discussed this mixed style, which was nevertheless experienced as difficult.

“...It is my expertise to argue with the one involved, but also with the person in charge and the board: what does it mean if you don’t want the employee to resume his original function, because you want to guarantee the company processes. What is the employee capable of? What is his knowhow…” [Resp. 10, HR]

Early in the process they questioned, for example, how an employee could do their parts without harming their own health, or in which way an employee’s expertise could be made use of. Here, a principle of “tailor-made measures” together with a degree of “obligation” was applied. Striving for a balance was sometimes linked to higher values regarding how to be a good leader.

“…The responsibility reaches far. You are responsible for an appropriate function. And my personal aim, but that is not formal, is that I would like to see the employee function with some pleasure in his job (…). For that is the purpose. One has to find a balance together, but you cannot do it alone…” [Resp. 10, HR]

#### Dilemmas during the trajectory

The complexity could be understood not only as a result of the different phases, requiring different communication styles, but also due to two types of moral dilemmas: balancing both the employee’s and the employer’s interests and dealing with an unfavorable diagnosis.

##### Balancing interests

Employers wished to strive for good arrangements for the employee and for the employer. But what counted more?

“…If an employee does not deliver and you do pay him—that is purely business—then it must be made debatable and if there is no change, well, then there are consequences. So that is the dilemma. On the one hand you understand but on the other hand, if you have been absent for one or two years and if you are not back on the former level, then there is a financial component. And I find that difficult…” [Resp. 21, LM]

Some moral problems confronted the employers with the question to what extent a business-oriented attitude was required and how much attention had to be given to the employee’s needs.

“…Even with the best intentions to position somebody, that makes it difficult. Often you want a lot, but it must be workable. And that workability is sometimes passed by too fast…” [Resp. 11, HR]

Balancing both needs faced them with a difficult reality: which were the most important matters to discuss and in which moment? The interviewees wanted to act carefully, and this brought up the question whether their decisions were well-balanced in an ethical sense.

“…How I approach people with cancer, well, that is ethics. That is looking for the right thing for someone else. And what is right in that case? And when it is right for the other one, it should also be right for the organization. So that is the dilemma that occurs. And sometimes it can be a moral dilemma, for which of the two is given most weight?...” [Resp. 8, LM]

Showing empathy, making RTW plans, and meeting the demands of the company had to be combined. The employers said that they searched for the right moment to discuss the important matters. For example, when does employability come up for discussion? Was it after treatment, or at the employee’s initiative? Not all employees were able or motivated to return to work after cancer. How to adapt the tasks to the employee without disadvantaging the companies’ interest was another dilemma in this respect. To what extent were they allowed to make demands and push the employee’s boundaries?

##### Dealing with an unfavorable diagnosis

The interviewed employers especially experienced a moral dilemma when the employee’s condition got worse because of an unfavorable prognosis. Could they confront the employee, who might want to stay active, with the fact that he or she did not perform well enough anymore? Were they allowed to take away responsibilities from the employee concerned?

“…And that is a huge problem, as an employer it gets you to the point where you think: well, I don’t want to send him home because I cannot do that to him, but I can’t keep him either…” [Resp. 7, LM]

Towards the end of legal sick leave payments, one has to apply for disability pension if not working. Employers struggled between requiring a needless procedure and a judgment of being unfit for work for their terminally ill employee and extending the payment for sick leave beyond 2 years, thus putting a burden on the organization’s financial resources.

“...He has already had a terrible message: yes, you have cancer and you are going to die. Look, according to the law we would have every right to say: ‘we will ask for early examination’ (…) but well, should you give someone who has been, well, let’s call it sentenced to death, give the feeling (…) ‘you cannot be of any use to us’? Luckily we have chosen not to act this way and to keep him employed and let the process take its course (…) As employer of a big company you should be ashamed to do so…” [Resp. 4, HR absence coach]

Employers sometimes felt caught between their sincere concern for the employee and the professional realism demanded of them, but they varied in dealing with these moral dilemmas. Communicating carefully and transparently was seen as the most important thing to do.

“…Did we, as employers, do the right thing for you? Do you have the feeling that we missed chances that you would have been able to work and we did not take the opportunity? And the other way around: did you, according to your own feeling, do the right thing? Did you perhaps miss chances and can we facilitate you in that? Those are our most important questions…” [Resp. 9, HR]

#### Differences between employers, employees, and organizational cultures

We noticed large variations between employer experiences in this study. These variations can be understood as stemming from the personal differences between employers and between employees and differences at the level of the organization.

##### Differences between employers

Employers personally differed regarding experience with cancer, RTW, and communication. For instance, employers having private or professional experience with a cancer diagnosis felt more prepared to deal with the employee’s despair.

“…I think it has to do with a form of emotional maturity, experience, life experience with close confrontation. And that it can be useful when you have to respond to people or when you want to offer help in the field of cancer. I, for sure, would not have been able to do it some years ago (…). It does help when you have some life experience and know a little about the impact of cancer. In all modesty I think I know something about that, yes…” [Resp. 4, HR absence coach]

Further, employers had different levels of experience with guiding RTW and, in addition, had different personal opinions regarding the approach to and speed of RTW.

“…That is up to the professionalism and the expertise of our organization, to persuade an employee. And I have the idea: there you never stop learning…” [Resp. 3, HR]

“…For with cancer you actually get your death notice. So you should handle it very carefully, ethically I think, extremely accurately. Also to the supervisory board. Don’t let yourself be pushed into a role that doesn’t fit you. But that requires courage. And that requires maturity, that requires seniority…” [Resp. 10, HR]

Some wanted to spend a lot of energy in standing up for and protecting the employee, especially in case of an unfavorable prognosis. Finally, employers had different amounts of experience with communication and reported feeling more or less comfortable in terms of communication skills.

“…I feel privileged that I can do this (…) I am glad I have that expertise and that I am not afraid of that. And that I find it important to just do well concerning the human component and the business. That that can be done in a balanced way…” [Resp. 10, HR]

##### Differences between employees

According to the employers, the individual employee’s medical situation, attitude, and motivation also played a role.

“…We had the idea: if you just show us that you think proactively, then we will do the same…” [Resp. 29, HR]

Cancer prognoses differed widely and were unforeseeable. The employers took into consideration how the employee’s attitude was appreciated at the workplace, whether the person performed well before the cancer diagnosis and what the person was like.

“…And I want to enjoy such a person with such a positive attitude for as long as possible, so I look for what is possible. And as long as working here gives meaning to a person’s life we must try to continue…” [Resp. 17, LM]

The employers varied in taking the employee’s attitude into account during the trajectory. They might for instance feel forced, as it were, to act in a more business-oriented way with regard to available work options when there was no RTW initiative taken by the employee.

##### Differences at the level of the organization

The role of organizational policy and company size was also felt. Generally, employers felt that the existing procedures had to be followed. However, organizations differed regarding strictness of RTW procedures. For example, organizations with more lenient rules offered more room for employers if, in their eyes, a mild approach was asked for in the concrete situation.

“…With the procedures [within the company] we can go many ways. And, in my opinion, that also should be flexible, that you can adjust to the situation, for not one person is the same, not one disease is the same and not one approach people have is the same. So, in my view, tight procedures would only obstruct you in that…” [Resp. 22, LM]

Degrees of strictness could be understood as a product of the type of work being done and the economic resources of the organization. For example, the interviewees from a hospital seemed more open to a more lenient approach because they all highly valued caring for the employee but also because the size of the organization meant that financial consequences were felt less. Further, organizational cultures differed, offering different degrees of latitude to make decisions on the RTW process to line managers and HR managers. In line with the Dutch law, accountability for the sickness absence and RTW process could be given to the line manager or the HR manager. In general, line managers felt connected more closely to the employee concerned than HR managers, but we found that the differences between the different employer representatives were mainly related to their experience and skills (rather than to their position in the organization). Differences between private and public organizations could not be found.

### Employer needs

Communication was regarded to be pivotal and in line with that employers reported the need for more advanced communication skills. Further, the trajectory was experienced as complex and requiring more experience and knowledge than employers often had, so knowledge and experience were regarded to be necessary. The trajectory involved decision-making that brought up many dilemmas, and some employers referred to a need for support in ethical decision-making.

“…Often you need courage to do things. Or just the courage not to. And to see the difference you need a lot of wisdom and you need to be somewhat older, I think…” [Resp. 8, LM]

Finally, the Dutch legislative framework was considered helpful only during the first 2 years, indicating a need for examples of how to deal with longer cases. In case an employee passed away, care had to be given to their close family as well.

#### Communication skills

Employers would like to become more skilled in communicating both about the cancer diagnosis and the employee’s employability. Some employers felt uncomfortable or caught between rules and feelings, especially when they had little experience in this matter.

“…It’s just, how do you deal with people with cancer, that’s another thing. After all, it depends on the contact you have with your colleague, that can be difficult sometimes, or hard, which is in conflict with the process of going back to work, at least that is how it feels…” [Resp. 19, LM]

According to the employers, the employer-employee connection was crucial. The employers wished to work together to understand each other’s attitude and situation and to make progress in the RTW trajectory. They found that it was an extra difficulty to combine both the cancer diagnosis and the employability in the discussion.

“…And then it is groping, searching, very carefully, about how you are going to lead this conversation and then sometimes you notice that you lack the basic skills (…) Then, based on the findings of the company doctor, you start the conversation and then I consult the case manager [HR] to ask him to assist in this conversation and then you do it together…” [Resp. 19, LM]

According to the employers, they had to be receptive to the employee’s needs. They also had to assess the employee’s availability, taking into account the medical situation. To address this difficult situation, separate appointments were sometimes planned: one about the illness, the other about the employee’s employability.

“…Yes, we really had two conversations. Of course we talked about the disease and the other conversation was about job performance. And then you wear two hats. It is about the availability based on the activities he has to do. But you can’t, in that same meeting, say: ‘okay, that is done. Now then, what about your illness’…” [Resp. 19, LM]

Further, an empathetic and proactive, but patient attitude was felt to be a necessary condition to manage RTW to everyone’s satisfaction. Employers mentioned that it was demonstrably difficult to guide a close-lipped employee or an employee who strongly mentally suffered from the cancer diagnosis. Lack of openness put a severe strain on the employers and raised serious obstacles in the trajectory. This could lead to essential information being missed.

Some employers were not capable of dealing with the employee’s emotional situation. Anger and grief are part of dealing with the diagnosis or an approaching death in the eyes of the interviewees. This could lead to friction when discussing RTW alternatives and adaptations.

“…And I say: I know exactly what goes wrong. I try to convince you of something and ‘you don’t get it’. And you try to convince me of something, but I don’t get it either. So I think we better leave the contents and concentrate on the process, for that is what it is all about…” [Resp. 29, HR]

The employers tried to deal with this, especially in case of a severe prognosis. How should they manage the situation personally?

“…Yes, I have bad nights every now and then. Then I say to my wife: I’ll just have a lousy day today. X [ill employee] is not doing well, and so on…” [Resp. 22, LM]

“…The problem with cancer is the sadness you have to cope with; I could talk about it at home. Well, that is quite something, for things can be heavy for a manager…” [Resp. 15, LM]

#### Knowledge and experience

The interviewed employers expressed having unsatisfactory basic knowledge on the divergent cancer prognoses and their effects on employability, also in the long term. They felt dependent on the employee’s openness regarding the medical situation.

“…Without having to reveal your medical secrets. But that you actually explain, what is cancer, what does chemo do to you. That information, mutually, that is crucial…” [Resp. 11, HR]

As they were legally not allowed to ask for the nature and origin of the diagnosis, employers felt obliged to respect the employee’s privacy. The occupational physician is not allowed either to provide the employer with medical information. This was experienced as difficult as it could make deciding on employability harder for the employer.

#### Ethical decision-making

At times, the employers did not really know whose interest counted more. Taking into account all relevant factors, dealing with both interests was experienced as extremely difficult.

“…How do other employers cope with it? Do they struggle with it as well? And what is finally decisive to do or not do things? And do they consider ethics or is it like: ‘no, we are cruel and only consider the financial side. Knock, knock, knock, this is the Gatekeeper’s Law and here it all ends…?’ Or are you really concerned about the impact for the employee with cancer and its consequences and do you decide on that basis? For that is considerably different…” [Resp. 4, HR absence coach]

Some expressed their wish to be judged as a good employer, but they truly wrestled with ethical decisions that had to be made.

“…At a given moment, when two years have almost passed by, it is clear: ‘this is the path, this is what it will be like’. That is somewhat theoretical, but anyway, it depends very much on the course of things. For when someone really reaches the terminal phase, then to me it is an inappropriate form of communication. It also depends on the relationship you have with your employee…” [Resp. 23, LM]

#### Examples of how to deal with “the end of the two years”

The RTW law provides clear rules, which gave structure to the process. However, the employer had to focus on the step-by-step-plan for the 2 years of sick leave required by law, because of the strict rules in place. All actions and adjustments had to be reported strictly and carefully: “without lying and in as favorable a way as possible for all parties” [Resp. 10, HR]. It was also reported that tailor-made measures were difficult to realize and report.

“…On the one hand I think it is good to have guidelines and they are well abided by, but on the other hand it is a corset and I think: I miss customization (…) for you are tied to the protocols and a limited time to reintegrate someone…” [Resp. 27, LM]

From the employers’ point of view, the transition to social insurance, after 2 years, was experienced as stressful for both the employee and the employer, because of the financial impacts. The timing could also be inconvenient.

“…Then he worked for 10 or 16 hours a week and at some moment we thought: well, you have to get better; if not we get to two years of illness. And then what…” [Resp. 29, HR]

“…Because it remained dim and vague what the hour load could be in the end, at a given moment it became a case of boarding up the file, that, as employer you undertake all necessary actions, in order to avoid the obligation to continue paying wages…” [Resp. 11, HR]

The employers wished to share best practices about RTW of employees with cancer and to feel assisted when having to deal with concerns and dilemmas.

## Discussion

**Fig. 1:**
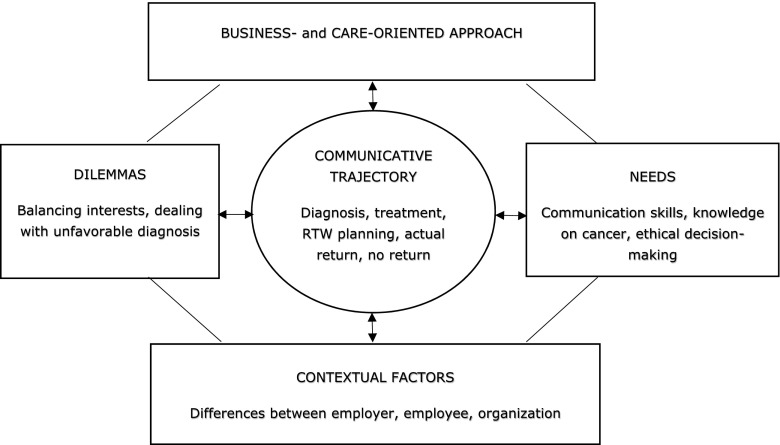
Employer experiences and needs

The aim of this study was to offer insight into Dutch employers’ experiences and needs regarding return to work (RTW) of employees with cancer (Fig. 1). The employers interviewed reported having a strong sense of responsibility to support their employee with cancer. This showed itself in an intense, complex, and continuous communicative trajectory from the moment of diagnosis until either the RTW or the employee’s passing away.

The employers followed the law, but the law is not sufficient to shape this complex trajectory fully. Some employers tended to emphasize the personal interest of the employee, others the interest of the company or employer. Some even felt an ethical obligation to balance these matters, as they considered this to be expected from a good employer. Employers faced an ethical challenge as a result of the dilemmas arising from balancing both parties’ interests and supporting people with an unpredictable cancer diagnosis. The interviews revealed that employers demonstrated a willingness to achieve suitable RTW options, but, generally, they expressed a need for better communication skills and more information on cancer to be able to negotiate the dilemmas they experienced. Given the strict Dutch legislation and positive bias of employers (see the “Strengths and limitations” section), it was surprising that the interviews were so diverse in approach and tone. This appeared to be closely related to employer and employee characteristics, and to the organizational culture. The different factors involved confirmed the complexity of the trajectory experienced by the employers themselves.

The complexity of RTW following a period of work disability has already been acknowledged in a Canadian setting [21]. Besides the different employer, employee characteristics, and organizational cultures described, which add to the complexity, other components have also been found to be influential (e.g., workplace support and job demands, access to health care), which confirms that RTW cannot be simplified [21]. An Australian study pointed at the complexity and communication difficulties in the staged RTW process. Keeping in contact with employees struggling with illness was a way to offer assistance [14]. Yarker et al. [22] highlighted the importance of communication within the workplace and the need to provide better support to line managers. Dewa et al. [23] reported that even workers’ communication skills should be trained so they would be better able to communicate the effects of treatment of their cancer on their ability to work. Egan et al. [24] mentioned the need for improvements in communication to empower people with cancer. Our study offered further insight into the process of communication between the employer and the employee, which helped to understand and confirm the importance of a proper, intense, and differentiated communication during several stages in the RTW trajectory. Although many of the employers interviewed were highly experienced, they still faced dilemmas and needs for support while supporting their employees.

Regarding the different approaches, our aim was to contribute to a better interpretation of the employers’ experiences and not to judge whether a business-oriented or a care-oriented approach is better. Emphasizing one aspect might lead to a one-sided representation of supporting an employee with cancer. However, it turned out to be difficult to combine both parties’ interests. The ethical responsibility is perhaps expressed in finding a balance or trying to do so. The appropriate way to support an employee might change during the trajectory, per employee, and across organizational contexts. The approach should be tailor-made, if possible, but employers had to deal with external factors in this respect. Searching for a balance was found to be a strenuous experience. When solving conflicts, employers tended to contact the occupational physician to decide on conflicting RTW options. McKay et al. [14] saw a role for an independent third party, a mediator, to help weighing the interests. Providing training for employers (especially with regard to communication) was also recommended by Johnston [25]. Being able to deliver sensitive information seemed to be especially important. This might relate to the difficulties the interviewed employers experienced when discussing illness and work issues or when confronting the employee with their inability to fulfill the tasks expected of them adequately.

The lack of medical knowledge regarding cancer, including the ability to deal with the uncertainty of the prognosis, coincides with one of the themes identified in RTW after stroke [26]. Recently, Dorland et al. [27] concluded that it is important to enhance knowledge about cognitive symptoms to support cancer patients and improve their functioning. Among others, Amir et al. [10] elaborated on the burden experienced in RTW support following cancer. In line with our results, some participants in this study preferred to support cancer survivors rather than persons with psychological problems. This might stem from the premise that cancer just happens to you and a person cannot be made responsible for having cancer. It is perceived to be a legitimate diagnosis.

Regrettably, more than 15% of the employees discussed passed away during the trajectory. Although it was not discussed openly, it was felt that many employers seemed to approach a cancer diagnosis as a life-threatening disease. Awaiting the treatment result after learning of the disease could also be understood from their own concern and expectations regarding RTW of the employee in question. We saw that employers did their best to help returning employees in carrying out their work in a healthy way. In line with Jensen et al. [28], employers absolutely respected the employee’s absence during treatment. Although cognitive and social problems are reported to have a negative impact on RTW after cancer [29–31], the employers we interviewed hardly mentioned these as barriers to starting the trajectory of their employee. In general, they relied on the occupational physician and his/her conclusions and tended to act accordingly.

In the Netherlands, there is a legislative framework with clear guidelines and a focus on supporting RTW of sick-listed employees (http://www.uwv.nl/overuwv/english; https://www.government.nl/ministries/ministry-of-social-affairs-and-employment) [18]. In this study, experienced employers mentioned that they might not need the legislation anymore; they knew how to deviate from the dictated steps, in consultation with other parties involved (e.g., the HR department or the occupational physician). They might say this because they seem to have internalized the legislation, similar to the internalization of rules found in Belgian employers one decade ago [32]. As such, the legal framework was used as a facilitating leading principle and not as a checklist or a procedural duty to fulfill. However, legislation seemed not to be enough to support employers in carrying out their full responsibilities in the RTW trajectory. Many employers were deeply involved with the employee and wanted to take care of them beyond the 2 years demanded by law. It appeared, therefore, that the Dutch legislation stimulates communication about RTW from an early stage, directly or indirectly. A lack of such legislation might lead to neglecting the needs of cancer survivors in relation to RTW [12].

### Strengths and limitations

Rich data enabled us to illustrate the complexity of an RTW trajectory after cancer and the challenges that employers were confronted with. The fact that the research group and the interviewer were experienced qualitative researchers led to qualitative, rich, and robust interview data. After presenting the first results, a peer debriefing was organized and experienced researchers commented on the findings. Together with the use of QUAGOL [20], this contributes to the trustworthiness and the theoretical generalizability of the findings.

The interviewees described more than 60 employees (*n* = 63) involved in a RTW trajectory after being diagnosed with cancer. They described many difficulties they experienced, and more than 15% of the employees discussed died within 2 years (*n* = 11). A large variety of cases, with different professions, different cancer diagnoses (as well as different levels of severity), and different RTW outcomes, were included in the study. This enabled us to elaborate on the employers’ challenges in more detail.

It has to be emphasized that the study must be interpreted with caution. It is based on convenience sampling more than on theoretical sampling. However, after 14 interviews with HR managers, who had positive experiences, we searched specifically for line managers who had difficult experiences. We could only include the employers and organizations that were willing to participate. Nevertheless, these participants gave us a good impression of the way things are done when supporting an employee with cancer, including the needs employers themselves were confronted with. We did not find any employers of small enterprises to include, while large groups of Dutch employees work in such companies. These employers struggle with the financial burden of continuing to pay sick employees for 2 years. We did not stumble across organizations with “precarious work,” decreased employment security, inferior levels of pay, and no access to health and welfare benefits. And although we purposefully searched for diversity in terms of position, personality, experiences, and RTW cases, a selected group of employers was interviewed. Despite these limitations in employer variety and positive bias, we found large differences. This suggests that the variety among all Dutch employers is even larger.

### Implications

More research on the perspective of employers is needed, as this is one of only a few studies done so far. Small companies should preferably be included, as the burden of expenses (continuing to pay the employee and possibly paying for replacement) may weigh on them more heavily. But perhaps employers should also explore other priorities than financial ones in their organization. The concept of ethical leadership in relation to RTW, where integrity and trust are important values, should especially be explored further [33]. In a study about head and neck cancer, employers were recently advised to employ core human values such as compassion, empathy, honesty, and respect [23].

As a practical matter, the findings suggest that employers who are confronted with employees with cancer can take advantage of legislation that stimulates communication, planning, and work adaptations. Second, employers’ communication skills should be improved, so they are better able to sense when to bring up relevant themes, especially employability, and to balance both parties’ interests (employer and employee). Third, we advise helping employers to develop ethical leadership to make them feel better equipped to weigh up all the dilemmas they face. Fourth, an informative tool could be developed to fill an existing gap regarding the lack of knowledge on cancer and its consequences for work. Fifth, a way to exchange information between employers who have experience with this matter, such as a secure forum to share best practices (regarding ethical dilemmas, dealing with the law, balancing interests), would be valuable to better prepare employers for the complex RTW trajectory.

## Conclusion

Our description of the difficulties employers experienced offers insight into some of their needs, and helps us understand how they could be supported. Their difficulties related to matters of communication and balancing interests, as well as knowledge on cancer and the consequences for work. Transparency and clearness in employers’ organizations are needed to find creative but legal solutions, together with enough room to handle things in accordance with their own views. It is also advisable that employees discuss their wishes regarding disclosure, communication, and work issues. The complexity of the RTW trajectory requires more than a legal framework and organizational guidelines to be able to give optimal RTW support, tailored to every specific situation. In conclusion, we recommend developing an informative tool to help employers in supporting their employees with cancer in the RTW trajectory.
